# Establishment of a Framework to Support Multi-Faceted Initiatives for Pharmacy-Practice Transformation: Lessons Learned

**DOI:** 10.3390/pharmacy9030153

**Published:** 2021-09-08

**Authors:** Renee Robinson, Shanna K. O’Connor, Hayli Hruza, Elaine Nguyen, Michael A. Biddle, Angela Jaglowicz, Thomas G. Wadsworth

**Affiliations:** 1 UAA/ISU Doctor of Pharmacy Program, College of Health, University of Alaska Anchorage, Anchorage, AK 99508, USA; tomwadsworth@isu.edu; 2 College of Pharmacy, Idaho State University, Pocatello, ID 83209, USA; shannaoconnor@isu.edu (S.K.O.); elainenguyen@isu.edu (E.N.); michaelbiddle.pharmd@gmail.com (M.A.B.J.); 3 Whatcom County Health Department, Bellingham, WA 98225, USA; hayli.hruza@gmail.com; 4 INTEGRIS Health, Oklahoma City, OK 73112, USA; angela.jaglowicz@integrisok.com

**Keywords:** practice transformation, health services, medical-billing, payors, pharmacist

## Abstract

We describe the first two years of a multifaceted, five-year program to support sustainable pharmacist-provided health services in Alaska. In 2018, the Alaska Pharmacists Association funded the Sustainable Education and Training Model under Pharmacist as Providers (SETMuPP) to train and support pharmacists to navigate the insurance medical billing process for nondispensing healthcare services. The SETMuPP employed a three-pillar implementation approach: (1) training and practice support infrastructure, (2) PharmD curriculum augmentation, and (3) advocacy and legislative support. The first two years have demonstrated the effectiveness of triad partnerships between professional associations, state policy makers, and academic centers to catalyze meaningful practice transformation.

## 1. Introduction

Pharmacists, the third-largest health profession in the United States (US), remain among the most accessible and underutilized healthcare providers nationally [1,2]. Today, many pharmacists provide healthcare services unrelated to dispensing, such as medication management, chronic disease management, transitions-of-care, and preventative care. These services are often provided in community or primary care settings and, in part, improve access to primary care services that are convenient and cost-effective [2,3,4,5].

However, despite the evident impact and success, the number of pharmacists providing these services in the private sector is relatively small, largely due to legislative, regulatory, and resource barriers. These barriers are complex and have led to unfair and inequitable reimbursement models that impede practice sustainability [6,7,8,9,10].

In 2018, the Alaska Department of Health and Social Services (DHSS) Section of Chronic Disease Prevention and Health Promotion (CDPHP), received funding under a five-year cooperative agreement with the Centers for Disease Control and Prevention (CDC) to support diabetes and cardiovascular disease prevention and management efforts. The CDPHP subsequently partnered with the Alaska Pharmacists Association (AKPhA) to mobilize Alaskan pharmacists to engage in diabetes and cardiovascular disease prevention and management efforts. As a result, the Sustainable Education and Training Model under Pharmacist as Providers (SETMuPP) program was created.

The CDC, DHSS, and Section of CDPHP’s primary objective is to better manage costly, chronic health conditions such as diabetes, heart disease, and stroke [11]. Consequently, the ultimate mission of the SETMuPP is to mobilize community and primary care pharmacists in Alaska to address the current gap in access to and coverage for diabetes and cardiovascular prevention and health management services through education and the collection of data and information to support advocacy efforts. In order to achieve this mission, SETMuPP established a model for pharmacy practice transformation to: (1) provide the necessary training and support for pharmacist-providers to navigate the established insurance medical billing processes and (2) advocate for policies and regulations that support fair reimbursement for pharmacists providing those healthcare services. The focus of SETMuPP was not on the expansion of pharmacist scope of practice, but rather, on the fair and equitable reimbursement by payors for services already within the scope of pharmacy practice in Alaska.

The SETMuPP program was designed through a contracted collaboration between AKPhA and University of Alaska/Idaho State University (UAA/ISU) Doctor of Pharmacy program. The partnership with UAA and ISU College of Pharmacy represents the only in-state Doctor of Pharmacy program in Alaska and is an academic center for the profession. Idaho is also among the most progressive states for pharmacy practice, with an expanded scope of practice and progressive legislation and regulations that support insurer reimbursement for a number of direct patient care services. Core members of the SETMuPP team are UAA/ISU faculty, as well as AKPhA leadership and the Alaska Board of Pharmacy, and medical billing consultants. To achieve its mission, SETMuPP employed a three-pillar implementation approach: (1) training and practice support infrastructure, (2) PharmD curriculum augmentation, and (3) advocacy and legislative support. The training and practice support arm focuses on establishing practice pilot sites and developing an insurance medical billing resource toolkit. The curriculum augmentation arm works on developing, implementing, and assessing medical billing training within the curriculum of UAA/ISU Doctor of Pharmacy program. Finally, the advocacy and legislative support arm focuses on researching state laws and insurance policy, convening stakeholders, and developing advocacy support materials for AKPhA. The intent of this article is to summarize the actions and findings of the SETMuPP program in the first two years of funding, which we hope will provide a framework for other states and associations to pattern after.

## 2. Accomplishments

### 2.1. Pharmacist Training and Practice Support

AKPhA and the SETMuPP team partnered with the DHSS’s Diabetes, Heart Disease, and Stroke Prevention Program to conduct focus groups and semistructured interviews to identify barriers related to coding, billing, and reimbursement for services provided by outpatient pharmacists. Three high-level themes believed to impact the uptake of nondispensing pharmacist services were identified in the qualitative data: training needs, resource needs, and system implementation needs. Gaps in knowledge regarding existing and potential nondispensing pharmacist services were noted among all three stakeholder groups. Most were unaware of the different types of services pharmacists are capable of providing, the extensive training pharmacists have, and the potential cost-savings associated with pharmacist-provided health services (e.g., immunizations). These results were used to develop and conduct a statewide pharmacist survey to identify and better quantify the barriers that pharmacists face when providing diabetes and cardiovascular disease prevention and management services. The questions referred to the number and type of direct pharmacist-provided health services distinct from traditional prescription dispensing functions, the capacity to provide services, and the barriers to the provision of service. The services of interest included, but were not limited to, medication therapy management (MTM), collaborative drug therapy management (CDTM), specialized patient education and counseling (e.g., diabetes), immunizations, point-of-care testing, and those currently being provided. Finally, a SOAR (strengths, opportunities, aspirations, and results) analysis was completed. The results of our preliminary analyses suggested that pharmacists not only could benefit from additional training in coding and billing the medical benefit or nondispensing services, but also that additional legislative, regulatory, and technology supports could better facilitate the expansion of pharmacist-provided nondispensing services, especially in rural and underserved communities. These findings provided a framework to identify training needs that were used to develop a draft toolkit and training program. The goals of the toolkit and training program are to promote existing medical billing best practices and translate them to a pharmacy audience. The toolkit consists of established billing and reimbursement materials/resources utilized by all other health disciplines (e.g., credentialing and privileging process guidance, site-assessment checklists, contract drafts, collaborative practice agreements, billing forms, hands-on exercises for pharmacists to practice coding and billing encounters, and audit processes).

This toolkit, along with SETMuPP-delivered training, served as the basis to educate staff at two primary care pilot sites, establishing a shared vernacular and understanding of service reimbursement process, and promoted “buy-in” from health system administrators. The toolkit and training were also more widely disseminated at a one-day AKPhA-sponsored program focused on medical billing for pharmacists. To assess the impact of the pilot project implementation and toolkit, SETMuPP team members routinely connected with the pharmacists at the pilot sites. Claim submissions and rejections were collected to identify and respond to additional training and support needs. Based on the experience at the initial pilot site, we identified breaks in the claim submission process and tailored workflows to address these communication and information sharing breakdowns. These lessons were used to develop a second iteration of the toolkit/training for the second pilot site, a Federally Qualified Health Center (FQHC). SETMuPP team members also created and deployed a patient satisfaction survey to assess community interest at the initial pilot site. Patient satisfaction scores were overwhelmingly positive, with a majority of patients expressing a high value of the services they received (publication pending).

**Figure 1:**
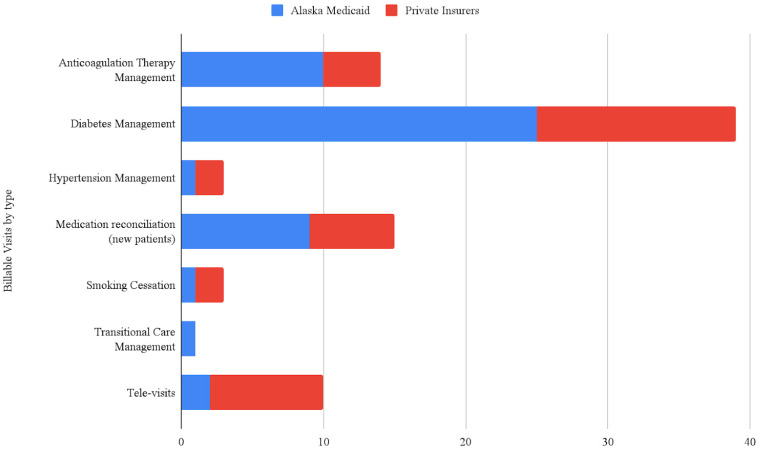
Billable visits by type and payer.

Services potentially eligible for billing under the medical benefit were delivered by both pilot sites. Claims were generated and submitted according to the usual practices at the site, thus site-specific billing personnel were responsible for claim submission and tracking. Service data was submitted by both pilot sites to the SETMuPP team to measure the quantity and type of services delivered. Figure 1 represents the billable services delivered from pilot sites 1 and 2 within the first 18 months, broken down by visit type and payor.

The toolkit will continue to be reviewed and updated to best meet the needs of different practice sites. In Years 3–5, the SETMuPP team efforts will focus on the unique training and resource needs of pharmacists practicing in the community pharmacy setting and adapting the resource toolkit to community pharmacy.

### 2.2. Integration of Billing into the PharmD Curriculum

**Figure 2:**
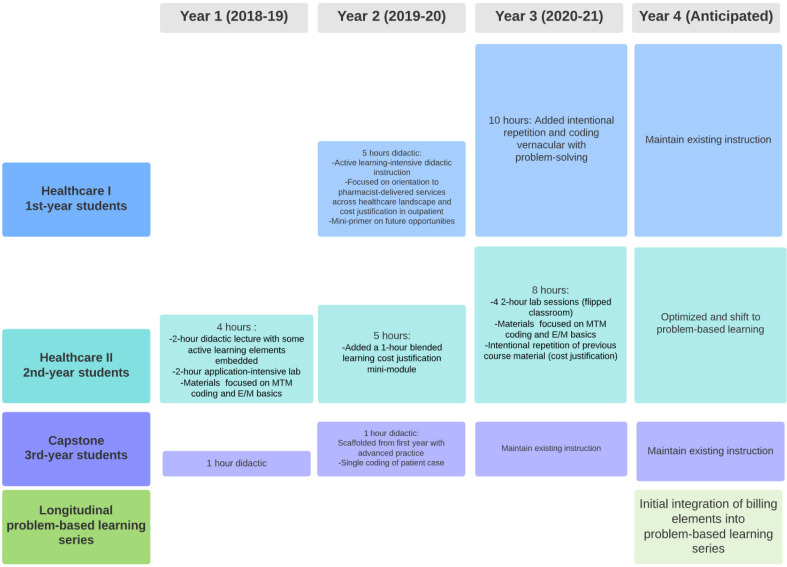
Integration of billing information in PharmD didactic curriculum Years 1–3, 2018–2019, 2019–2020, 2020–2021.

Before implementation (Year 0), the integration of billing within the curriculum was topical and found throughout the curriculum without intentional scaffolding. High-level overviews of medication therapy management (MTM) were provided without specific coding-based information. Specific information related to billing for dispensing services was covered via didactic lecture without application activities. Knowledge was assessed via examination and completion of billing case studies. SETMuPP efforts to integrate medical billing in all three years of the didactic curriculum across the project years can be seen in Figure 2.

Year 1 saw the first iteration of a core mini-module of didactic instruction and lab-based practice specifically focused on billing for pharmacist-provided healthcare services in the outpatient setting. Didactic session activities included the walk-through of a case with sample coding. The lab activities focused on students replicating the coding process with different cases in groups. Feedback was provided by on-site pharmacist facilitators with expertise in medical billing and coding.

Year 2 saw replication of the mini-module of billing and coding from Year 1 and expansion of the curricular scaffolding surrounding billing. A modified, one-hour version of the AKPhA workshop was presented to third-year student pharmacists in preparation for experiential learning. The information presented was designed to be an intentional repetition of the content this cohort of student pharmacists had been exposed to the previous year. Additionally, a foundational five-hour module was added and delivered to first-year student pharmacists.

Year 3 is underway and the original modules from Years 1 and 2 for second-year student pharmacists are being adapted. The material delivered to first-year students has been extensively remodeled so that students are exposed with more intention to the practicalities of insurance in the context of the US healthcare system and how the elements of the cost justification from Year 2 fit into said system. The second-year material has been reformatted to have an emphasis on in-class expert modeling and practice problem solving using a flipped classroom approach. Active pre-work is paired with lab-based independent problem-solving using a wider variety of patient cases. Capstone, the final didactic course in the Doctor of Pharmacy curriculum, will have a one-hour lecture reviewing medical billing and five embedded cases with live standardized patient interactions. The patient simulations are designed to interweave clinical, billing, and patient engagement skills learned throughout the curriculum. Overall, students and faculty support the additional training provided in the program.

### 2.3. Advocacy and Legislative Support

The advocacy core group conducted an extensive review of Alaska law and established two key findings: (1) pharmacists were already designated as billing medical providers in Alaska state statutes; and (2) delivery of many healthcare services was within the scope of practice for pharmacists in Alaska. From here, the team explored process issues impeding pharmacists from providing healthcare services and submitting claims to the medical benefit. Three major findings from this investigation were (1) pharmacists are already established as billing medical providers under Alaska statute and regulation; (2) pharmacists are not protected under current state provider antidiscrimination law; and (3) the Alaska Medicaid Portal arbitrarily excluded pharmacists from enrollment as providers due to a process error.

The advocacy group created a triad relationship between the UAA/ISU Doctor of Pharmacy Program, AKPhA, and Alaska’s pharmacy regulatory board. The intent of this collaboration is to unify the profession in the state by creating a shared vision, values, and vernacular. Through key collaborations, the advocacy core partnered with Alaska Medicaid to support enrollment of pharmacists as billing providers. Specific meetings were held with the state Insurance Commissioner to get their perspective and support. The Commissioner maintained that it would cost more money to the State to reimburse pharmacists for healthcare services, so they were initially unwilling to provide support for this project moving forward. This response parallels messaging related to attaining pharmacist federal recognition as billing providers under Medicare. Additionally, the Commissioner was unaware of pharmacists’ ability to provide healthcare services and associated the profession exclusively with dispensing activities. This response highlights the ongoing need at state and national levels to spotlight the nondispensing training and skills of the profession. The advocacy group collaborated with AKPhA to meet with legislators to discuss what pharmacists can do to improve access to cost-effective health services. In preparation for these meetings, individuals completed a one-hour training on “how to engage with state legislators”, which was developed and presented by an AKPhA lobbyist; received statistics supporting provider status and billing of the medical benefit; and learned about the SHARP educational support program for Alaska Health Care Students. These meetings helped to establish formal partnerships with the AKPhA legislative group and an action plan for the advocacy group to follow.

The action plan is ongoing and project plans for Years 3–4 are in place. The advocacy group provides ongoing support to recognize pharmacists as health care providers, to promote a shared vernacular, and to establish a unified vision for reimbursement and service access. In Year 2, the advocacy team and AKPhA legislative committee focused on implementation of the unexercised 2015 Senate Bill (SB51). This bill mandated that Alaska Medicaid add pharmacists to the provider-credentialing enrollment portal to support pharmacist billing of the medical and pharmacy benefit for pharmacist-administered vaccinations. However, the Medicaid Portal was not configured to enroll pharmacists as billing providers, creating a procedural “hard stop” for pharmacists seeking to enroll.

Having identified this underlying procedural barrier, the advocacy group contacted and collaborated with the sponsoring legislator of SB51 to identify paths forward and find solutions. The team compiled information in support of these necessary changes and provided these data to the DHSS Director and the Medicaid Pharmacy Program Manager. Data from the DHSS-funded naloxone project and the state Immunization Information System highlighted missed vaccination opportunities. The team consulted with Health and Human Services and Center for Medicare and Medicaid Services experts, who advised that the team focus on the Medicaid State Plan Amendment. The team requested that pharmacists be listed as “Other Provider” to ensure Alaska Medicaid receives matching federal funds. The team provided testimony and comments at the statewide Medicaid scoping meeting, advocating that Medicaid fully implement SB51 by adding pharmacists to the enrollment portal. The SETMuPP advocacy group’s momentum contributed, in part, to Alaska Medicaid finally adding pharmacists to the enrollment portal in June 2020.

## 3. Lessons Learned

The current medical billing processes used in the United States (US) are complex, multifaceted, and difficult to navigate for pharmacists without prior experience. It is further complicated by variation in state laws, interpretation of rules and regulations, different payor requirements, and information technology system barriers [10,12,13,14]. Adding to this complexity is the incongruence in terminology and lack of shared vernacular that impedes communication, dividing the profession.

### 3.1. Lack of Familiarity with Available Reimbursable Pharmacist-Provided Patient Care Services

Overall, Years 1 and 2 of the SETMuPP project demonstrated the need for improved collaboration between internal clinic billers, healthcare providers, leadership, and payers to capitalize on opportunities available to pharmacists within the healthcare team. Medical practices are often unfamiliar with the patient care services that pharmacists provide. The ability of pharmacists to bill for nondispensing services in a medical practice requires administrative support from the Chief Executive Officer and Chief Financial Officer, who are often unfamiliar with the coding and reimbursement of pharmacy patient care services. Partnerships with key stakeholders such as the Chief Medical Officer and other healthcare providers aid in the development of relationships such as collaborative practice agreements. Orientation of clinical and administrative staff—including nurses, medical assistants, schedulers, information technology experts, coders, and billers—is critical to ensure all parties are aware of what services pharmacists can provide in the context of provider types they are familiar with. In addition to the usual clinic-based meetings, it is imperative to hold regular meetings and discussions with the administrative team and billing department that cover medical billing and coding for pharmacists to promote reimbursement success. Despite these meetings, it may take time (e.g., a calendar year) for the medical practice to successfully submit pharmacists’ claims for reimbursement. Once pharmacists are submitting claims, follow-up with stakeholders is key to ensure claims are transmitted to payors, reconciled, and appealed if necessary.

Claim coders and pharmacists share responsibility to ensure accurate representation of the service(s) rendered. In our experience, this was the most confusing step for billing personnel because they were not familiar with “pharmacists as rendering providers” or the nondispensing services provided by pharmacists. This confusion created inappropriate claim modifications such as National Provider Identification (NPI) number substitution for a different provider or under-coding of the visit. Clear communication, focused data collection, and regular internal audits of services will help to ensure the integrity of the claim submission process and cost-effectiveness of services provided.

**Figure 3:**
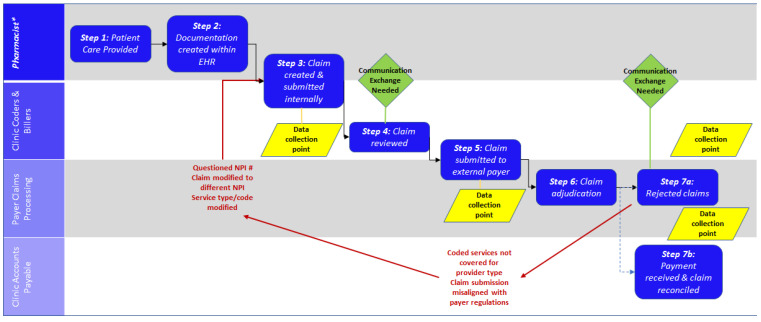
Claim process for services provided by a pharmacist.

In order to facilitate pharmacists’ understanding of the medical billing process, we have created a detailed flowchart. Figure 3 represents the claims process for pharmacists to be reimbursed for provided health services.

A pharmacist begins the process by documenting the encounter for the services provided (steps 1 and 2). Following documentation, the encounter is coded using International Classifications of Disease (ICD-10) and American Medical Association Current Procedural Terminology (CPT) codes. During steps 3 and 4, depending on the facility’s claim process model, the rendering pharmacist and/or coder will select, review and verify the encounter documentation codes selected. After codes are confirmed internally, the claim is “dropped” (i.e., submitted) to the external payor as shown in step 5. In step 6 and 7, the payor(s) confirm that plan-specific regulations and requirements are met, adjudicating or rejecting the claim. Typically, rejections or approvals are then communicated back to the clinic billing department. If the claim is rejected as demonstrated in step 7a, it is important for both the internal coder and the pharmacist to know why the claim was rejected. If the claim is accepted as demonstrated in step 7b, then it is important to capture reimbursement amount and reconcile this reimbursement internally within the facility.

As pharmacists expand their role in the provision of services billed to the medical insurance, close attention to data collection points (as indicated in Figure 3: type of services, rendering provider, CPT codes, the initial amount submitted, the reconciled amount received, and reasons for rejected claims) will help to facilitate internal quality improvement, cost-effectiveness analyses, and service long-term success.

### 3.2. Successful Advocacy Requires Awareness and Sometimes Small Changes

It is imperative that pharmacists have a baseline understanding of insurance laws and pharmacy practice laws that affect medical insurance reimbursement in order to advocate for the profession. Specifically, familiarity with how these laws intersect and align is critical, as is literacy with medical billing processes used by all other providers. Pharmacists must also be familiar with the bills being proposed by legislators and engage with policy makers and payors to effectuate existing law and promote the removal of reimbursement barriers that impede practice transformation. There is a need for supported positions related to lobbying specific to pharmacy, but as demonstrated by the SETMuPP model, progress can be made through the establishment of triad relationships, engagement of volunteers, and through small, targeted, stepwise initiatives.

### 3.3. Pharmacy Training Must Be Tailored to Specific Practice Setting and Site

Developing a comprehensive and effective training program is challenged by the heterogeneity of pharmacy practice sites and care delivery models. Pharmacists practicing at some practice sites (e.g., primary care clinics, FQHCs) may have more exposure to and experience with medical coding and billing than their peers at other sites (e.g., hospitals, skilled nursing, and community pharmacy). Additionally, the practice site may impart limitations based on insurance code and/or payment models that impact what services may be provided, which providers may perform the services, and how the services are coded and reimbursed. Even between similar practice settings, differences in the care delivery model employed by the practice site may further differentiate the roles and educational needs of pharmacists (and pharmacy staff). For example, a primary care practice that has integrated a pharmacist as a specialist for consultation will have different needs than a primary care practice that has integrated a pharmacist as a core healthcare provider. Furthermore, a pharmacist consultant embedded within a community pharmacy will have different needs than a pharmacist consultant embedded within a primary care clinic. It is important to clarify site-specific and care delivery-model-specific expectations early on in the process to ensure everyone is working towards the same goal and that pharmacist training and reporting needs can be identified and addressed.

## 4. Future Work

Lessons learned in Years 1–2 of this initiative have led to the establishment of necessary short- and long-term future work to promote pharmacist integration into the healthcare system as a billing provider, thereby increasing delivery of sustainable pharmacist-provided healthcare services.

### 4.1. Building a Shared Understanding of Pharmacist Services

As noted, other members of the healthcare team remain unaware of the many available nondispensing pharmacy services. These gaps require ongoing diligence in building a shared understanding with other members of the healthcare team as well as those who support healthcare delivery, such as billers, the executive suite, and information technology experts. To address these needs, the SETMuPP team plans to: (1) expand the number of participating community pharmacy sites in the upcoming year; (2) establish a bi-weekly training program for participating sites to work through implementation challenges and share resources; and (3) facilitate and support the AKPhA in advocating for the necessary regulatory and legislative changes to support reimbursement of pharmacist-provided health services by all public and private payors.

### 4.2. Expanding Opportunities for Pharmacist Medical Billing

Continuing education programming, such as the SETMuPP/AKPhA “Transformation Workshop”, must be made widely available to ensure understanding of the fundamental concepts and real-world application of coding and medical billing. The SETMuPP team will continue to create materials and resources and promote partnerships that deliver quality education to pharmacists, pharmacy staff, and student pharmacists. It is essential to create a practice-ready workforce through student pharmacist training. Schools and Colleges of Pharmacy need to train students to document, code, and bill for services before they enter the workforce.

### 4.3. Demonstration of Value and Information Dissemination

Quality improvement will continue to be data-driven, and the need for robust data collection and analysis across sites augmented to better support legislative advocacy efforts and promote widespread dissemination and sharing of lessons learned.

## 5. Conclusions

There is a clear, demonstrated need for states, schools, and the profession to join together to catalyze practice transformation. This transformation requires unified legislative action, intentional practice reform within practice sites, and evolving education models to train new and existing pharmacists. The SETMuPP model provides a framework for other states and associations to pattern after as they navigate their own initiatives related to pharmacist-provided healthcare services.
